# Optimisation of the ActWELL lifestyle intervention programme for women attending routine NHS breast screening clinics

**DOI:** 10.1186/s13063-020-04405-z

**Published:** 2020-06-05

**Authors:** Annie S. Anderson, Angela M. Craigie, Stephanie Gallant, Chloe McAdam, E. Jane Macaskill, Jennifer McKell, Nanette Mutrie, Ronan E. O’Carroll, Naveed Sattar, Martine Stead, Shaun Treweek

**Affiliations:** 1 Centre for Research into Cancer Prevention and Screening / Tayside Cancer Centre, Division of Cancer Research, University of Dundee, Level 7, Mailbox 7, Ninewells Hospital & Medical School, Dundee, DD1 9SY UK; 2 Physical Activity for Health Research Centre, Institute for Sport, Physical Education and Health Sciences, University of Edinburgh, St Leonard’s Land, Holyrood Road, Edinburgh, EH8 8AQ UK; 3 Department of Breast Surgery, NHS Tayside, Ninewells Hospital & Medical School, Dundee, DD1 9SY UK; 4 Institute for Social Marketing, University of Stirling, Stirling, FK9 4LA Scotland, UK; 5 Division of Psychology, School of Natural Sciences, University of Stirling, Stirling, FK9 4LA UK; 6 Institute of Cardiovascular and Medical Sciences, University of Glasgow, Glasgow, G12 8TA UK; 7 Health Services Research Unit, University of Aberdeen, Health Sciences Building, Foresterhill, Aberdeen, AB25 2ZD UK

**Keywords:** Breast cancer, Body weight, Physical activity, Lifestyle, Intervention

## Abstract

**Background:**

Around 30% of post-menopausal breast cancer is related to excess body fat, alcohol intake and low levels of physical activity. Current estimates suggest that there is a 12% increased risk in post-menopausal breast cancer for every 5 kg/m^2^ increase in body mass index (BMI). Despite this evidence there are few lifestyle programmes directed towards breast cancer risk reduction. This paper describes the process of optimising of the ActWELL programme which aims to support weight management in women invited to attend routine National Health Service (NHS) breast screening clinics.

**Methods:**

A feasibility study of a prototype programme aiming to change lifestyle behaviours was successfully undertaken. The programme used educational approaches and behaviour change techniques delivered by lifestyle coaches using individual face to face meetings and telephone sessions. To optimise the intervention for a definitive randomised controlled trial of weight management, data from the feasibility trial, focus group discussions conducted with the target population, feedback from the trial public advisory group and comments from peer reviewers were obtained. Concepts from implementation research provided further guidance to assist in the refinement of the intervention, which was then discussed and agreed by all investigators and the Trial Steering Group.

**Results:**

The results from the feasibility trial were considered appropriate for moving on to a full trial with 70% of participants finding the programme acceptable. The primary outcomes (weight loss and physical activity) provided an important focus for design input from the target group. The contributions highlighted the need to review programme duration, coach contact time, content and use of behaviour change techniques and communications generally (e.g. science and evidence, non-judgemental approaches and avoiding guilt). In addition, the need for emphasis on support rather than education became apparent. The recommendations from peer reviewers focussed on the magnitude of effort required to achieve the intended weight loss and weight loss maintenance. Implementation science supported the use of the capability/opportunity/motivation (COM-B)model in overall design.

**Conclusions:**

The optimisation process has facilitated the development and evaluation of a programme that enables the delivery of a promising intervention to achieve weight management in post-menopausal women.

**Trial registration:**

ISRCTN: ISRCTN11057518. Registered on 21 July 2017. Retrospectively registered.

## Introduction

Effective strategies to address the challenges of poor dietary intake, excess body weight and alcohol consumption and low levels of physical activity levels are urgently needed [1]. These health behaviours contribute to many non-communicable diseases including cardiovascular disease, diabetes mellitus and many cancers including breast and colorectal cancer [2–4]. Breast cancer is the most commonly diagnosed cancer in the UK, accounting for 15% of all new cancer cases [5]. Around 30% of post-menopausal breast cancer is related to excess body fat, alcohol intake and low levels of physical activity [6]. Current estimates suggest that there is a 12% increased risk in post-menopausal breast cancer for every 5 kg/m^2^ increase in body mass index (BMI) [7]. Despite this evidence, there are few lifestyle programmes directed towards breast cancer risk reduction [8].

Comprehensive approaches that tackle both population-level factors (e.g. policies such as minimum unit pricing for alcohol) and individual-level actions are desirable to achieve changes in health behaviours [9]. Lifestyle programmes can form an important component of such strategies and there is a need to develop and test evidence-based interventions that are effective and culturally and socially relevant to target populations [10]. Many lifestyle trials fail to have a significant impact on health behaviours and questions have been raised about the theoretical bases, content, dose and duration of the interventions used [11]. It is recognised that the development and optimisation of such intervention programmes is often limited because of emphasis on (and costs of) evaluating impact, time limitations within trial protocols, complex trial procedures and research governance requirements.

However, the importance of investing in intervention design and feasibility work is now being recognised and is increasingly gaining support from funding bodies [12]. Feasibility trials of interventions are principally designed to help identify uncertainties about trial procedures (research design parameters) but also provide an opportunity to evaluate intervention programme design, content, delivery, acceptability and fidelity [13, 14]. Being able to identify what intervention components and which processes “work well” and “work less well” from the perspective of the research outcomes, delivery teams and participants is key to informing final intervention content [15].

The further development and refinement (optimisation) of interventions as they move from feasibility to a full trial provides an opportunity to consider the optimal approach to achieving key study outcomes. Development time allows the findings from feasibility work to be considered as part of a re-appraisal of a programme including considerations of how the content relates to aims, how known moderators of behaviour change are incorporated as well as practical constraints and opportunities. Finally, and perhaps most importantly, programme engagement, delivery and content need to be guided by public (patient) perceptions, experiences, stated preferences and recommendations.

The aim of the current work is to describe the systematic process that was undertaken in the development of the ActWELL intervention programme in order to understand the changes made to the intervention between feasibility and the definitive randomised control trial (RCT) of weight management in post-menopausal women.

The full trial was funded as part of the Scottish Government Cancer strategy [16] to support cancer prevention. The Scottish Screening Committee will carefully consider the trial and, if successful, assessed as cost effective, and able to be implemented within the breast cancer screening programme, explore wider roll out. The UK charity Breast Cancer Now were key partners in the programme and were responsible for recruiting and managing volunteer lifestyle coaches to undertake bespoke training to implement the intervention programme. Due to increasing concerns over the potentially causal relationship between excess body fat and breast cancer the trial focuses on weight management and physical activity. The trial aimed to assess the effectiveness and cost-effectiveness of a theory-based, community delivered, minimal contact, weight management (diet, physical activity and behaviour change techniques) programme (ActWELL) over 12 months. The target for weight loss was 7% of body weight (based on the successful US diabetes prevention programme) [17]. Women with a BMI > 25 kg/m^2^ who were invited to attend routine breast cancer screening clinics (ages 50–70 years) were eligible for inclusion [18].

## Methods

Four sources of data were used to inform the design of the final ActWELL programme for the definitive RCT; the findings from a feasibility trial, focus group discussions with the target population, feedback from the ActWELL public advisory group and feedback from peer reviewers. The research team then reviewed the approach with respect to current concepts in implementation research. A protocol was drafted by the trial management group, presented and discussed with the investigators’ team resulting in some minor amendments and finally approved by the Trial Steering Committee.

### Data source 1- the feasibility trial

A full description of the formative work undertaken for the feasibility trial [19], procedures and outcomes are presented in detail elsewhere [18]. In summary, the programme was originally tested as a feasibility trial of lifestyle change (diet, alcohol, weight management, physical activity) over 3 months. Most (71%) of the participants were overweight or obese. The programme invitation was offered within the breast screening service in two National Health Service (NHS) sites. The feasibility trial was undertaken as a partnership between the research team, with endorsement from the NHS and several cancer charities and was funded by the Chief Scientist Office, Scottish Government.

The intervention aimed to help women attending NHS breast screening clinics increase physical activity, modify their diet, lower their alcohol intake and set weight management goals as appropriate. The measurement and intervention sessions took place in research institutions (rather than screening clinics). The programme was delivered by lifestyle coaches who were paid employees recruited for the trial and provided with a bespoke training programme. Participants received one face to face coaching session (1 h) plus six fortnightly calls for 3 months. The programme contents covered verbal and written information on lifestyle and breast cancer risk and personalised advice on activity, diet and alcohol with guidance on habit formation and relapse management strategies. Each participant was provided with a pedometer-based walking programme. The delivery was designed to be interactive, and evidence-based behavioural change techniques were used to motivate and support change including motivational interviewing, goal setting, coping planning and implementation intentions [20]. These parameters also formed the basis for the intervention phone calls that were planned to be 15 min in duration, and checked wellbeing, progress on implementation intentions and self-monitoring behaviours. Coach fidelity to the intervention protocol was assessed using transcribed audio recordings of a random sample of nine face to face coaching sessions, and scored in relation to the protocol.

Acceptability of the intervention components was assessed by an anonymous exit questionnaire given to participants after all study measures were completed. In addition, participants were invited to complete face to face semi-structured interviews.

### Data source 2- focus group discussions

Focus group discussions to inform aspects of the recruitment and delivery of the ActWELL programme for use in the definitive RCT were held with four groups of women ages 50–70 years with experience of being invited to routine breast screening clinics. These discussions enabled contemporary views on messaging credibility and acceptability of planned actions to be reviewed with women from across Scotland. Participants were recruited based on the social grade of the head of the household (ABC1 or C2DE). Overall, 27 women took part in the four focus groups (see Table 1). The sample is shown below:

**Table 1:** Focus Group Discussions - participants

**Group**	**Location**	**Social economic position of head of household**^**a**^ [21]	**Number of participants**
1	East of Scotland	ABC1	6
2	East of Scotland	C2DE	7
3	West of Scotland	ABC1	7
4	West of Scotland	C2DE	7

^a^Social grade was defined according to occupation of head of household [ref: http://www.nrs.co.uk/nrs-print/lifestyle-and-classification-data/socialgrade/]. ABC1 comprises managerial, administrative and professional occupations, and C2DE comprises skilled and semi-skilled manual workers and those who are not in employment

The first two groups involved two facilitators with one leading and the other supporting while the second set of focus groups involved one facilitator only. Discussion was initiated in both groups by asking participants to respond to the concept of being invited to participate in the proposed ActWELL programme. Women were shown mock-ups of flyers inviting them to find out more about the ActWELL study and asked how they would feel if presented with these while attending for routine mammography. Views on specific aspects of the proposed health messaging and overall programme were then explored using a semi-structured topic guide.

### Data source 3 - ActWELL public advisory group

A public advisory group (PAG) was established comprising three clients from screening attendees recruited by Breast Cancer Now, plus a patient advisor from the investigation team (chair of the public advisory group). The public advisory group was involved in every aspect of the trial including design, implementation, study conduct and data interpretation.

Draft study materials including recruitment posters, study invitation cards, intervention packs and accompanying leaflets were sent to the PAG for inspection and comments invited by email. All members replied.

### Data source 4 - peer reviewer feedback

The funders (Scottish Government) sought peer reviewer comments on all aspects of the trial, which the study team were invited to consider and to form a response.

## Results

### Feasibility trial

In the feasibility trial a pre-set recruitment target of 80 women from routine NHS breast screening clinics was achieved within 12 weeks and 65 participants (81%) completed follow up assessments at 3 months. The primary analysis showed significant between-group differences in body weight and (questionnaire-reported) physical activity measures.

The mean duration of the face to face coaching session was 90 min (range 65–130 min); the planned protocol time was 60 min. Mean duration of the telephone consultations was 22 min (range 10–54 min); the planned protocol time was 15 min. Full details are provided elsewhere [18].

The independent assessments showed that the intervention was delivered with high fidelity (close to the protocol). Deviations from the protocol included the coach setting goals rather than the participant, not discussing the intervention in terms of personal wellbeing and limited discussion of coping planning strategies.

Feedback on acceptability of the programme from exit questionnaires showed that face to face contact, telephone calls, educational materials, pedometers and topics raised in motivational interviewing were acceptable/very acceptable to at least 70% of participants. Goals (weight management, physical activity and diet) were set by more than 75% of participants but only 20% set an alcohol goal, largely because reported intakes were perceived as low. All goals were described as useful by 60% (and 80% of those who set an alcohol goal). Women rated the programme highly and 70% said they would recommend it to others.

Qualitative data obtained from 14 participants who took part in semi-structured interviews highlighted that information about the association between lifestyle factors and cancer risk was new learning. The coaches’ non-judgemental, positive approach was appreciated, and telephone contacts were highly valued. The pedometers were particularly valued by many women because they allowed participants to turn knowledge into experiential learning. One widely expressed view was that the programme should be longer in duration than the current 3 months. The changes made to the design of the full-scale trial made in response to these findings are presented in Table 2 (row 1).

**Table 2:** Changes in intervention resulting from preparatory work

Data source	Implications for RCT intervention	Changes implemented in full trial
Feasibility study	Feasibility intervention was effective in achieving changes in physical activity and bodyweight but not diet	Increase dietary guidance, personalised in line with national guidelines
	Additional face to face contact required	Provide two, shorter face to face visits
	Participants preferred extended programme and contact period	Increase programme to 12 months, enabling contact maintenance and longer-tern evaluation
	Intervention approaches identified as acceptable to participants	Retain acceptable intervention approaches: e.g. written educational materials and behaviour change techniques
Focus group discussions	The association between lifestyle and breast cancer needs to be clearer	Include current scientific evidence in coaches’ training to ensure they are fully equipped to respond to questions around breast cancer and lifestyle links
		Enhance infographics used in information packs and ensure reference links updated
		Reinforce association of lifestyle change with other positive health outcomes, including mental health
	Strong negative views about benefits of alcohol reduction	Embed alcohol messages with total caloric intake to introduce topic
	Ensure coaches appreciate, acknowledge and build on women’s previous engagement in lifestyle changes	Assess and comment on reported lifestyle changes
	Personalised advice is given to increase or maintain current physical activity	Be clear that discussion in physical activity relevant for all
	Importance of diet as well as physical activity needs to be clear, particularly for active but overweight women	Ensure clarity around importance of diet as well as physical activity
	Be clear about what the programme offers beyond education about physical activity and weight loss	Ensure coaches emphasise their educational and support role in personalised lifestyle change across a wide range of health dimensions
	Explore flexibility in appointments for intervention delivery	Provide flexibility in appointment times for participants, including evening and weekends
ActWELL public advisory group	Written and verbal communications should be inclusive and address current and future co-morbidities	Potential participants with low mobility may be screened out only if physical activity is contra-indicated for medical reasons
		Provide coaches with information on where to find links and assistance, as appropriate
		Written material should clarify concepts of risk reduction rather than prevention per se
		Ensure images for in-house materials are designed appropriately for this target group
Feedback from peer reviewer	Target weight loss of 7% is only likely to be achieved with greater dietary reduction than that used in the feasibility study	Enhance interactive learning on sugary drinks, snacks and portion control as these are relevant to excess calorific intake
		Consider including discussions about diet in both face to face sessions: Session 1 - Focus on snacking; Session 2 - Focus on total diet including portion sizes, meal choices, patterns and successful strategies for managing dietary intake
	The 12-month programme needs to take a weight management approach incorporating weight loss and weight maintenance	Advise coaches on weight loss maintenance, but this should be discussed with the research team on a case by case basis - especially if participants wish to continue weight loss
		Participants moving on to maintenance will be encouraged to monitor and record new habits using the ‘Ten Top Tips’ shown to be successful for weight loss maintenance over a 2-year period [22]
		Emphasise importance of regular phone call support by offering up to 9 calls during the 12-month period
	Additional support may be required to maintain adherence over a 12- month programme compared to that required for a 3-month programme	To encourage adherence, coaches should: - identify positive behaviour changes - give positive feedback - ask participants to report current body weight and provide supportive advice/comment
		Coaches’ training should include how to offer programme re-starts and revised goals for participants who have breaks in programme participation (e.g. illness, holidays)
	Learning from feasibility study on intervention session timings and improving fidelity	A detailed breakdown of the timing of programme delivery will be incorporated in coaches’ training including role modelling approaches, test timings and self-report of first five participants

### Focus group discussions

Several issues emerged from the focus group discussions, which had implications for the proposed intervention. Some of the findings were similar to those reported for the feasibility work [18] 4 years earlier and confirm key issues that remain to be considered in the intervention content. First, the association between lifestyle and breast cancer needed to be clearer. There were varying levels of awareness among women of similar statements in the draft invitation flyers about the links between lifestyle factors, particularly weight, and breast cancer risk. While some found the statements credible, others were sceptical, and perceived that breast cancer could develop because of genes recurring within families, that it was related to hormones or in some cases was “just your luck”:

*…a lot of people get breast cancer when they are slim, fat or medium. It doesn’t have to be because you are overweight. I have known a lot of people who have had breast cancer and they’re very slim.* (Group 2, C2DE)

Others perceived that following a lifestyle involving a healthy diet and plenty of exercise, and maintaining a healthy weight, was important for good health and avoidance of a range of health conditions regardless of whether they believed the evidence on a specific link with breast cancer.

In one of the second set of groups, where one of the draft invitations highlighted that 30% of breast cancers were due to lifestyle factors, a participant was shocked to hear the extent of these links:

*I was quite shocked to see 30% can be ... that figure there, associated with your lifestyle to breast cancer. I didn’t realise it was as high as that.* (Group 4, C2DE)

Second, messages implying that reducing alcohol intake could reduce breast cancer risk elicited strong sceptical comments with the potential to reduce engagements:

*What is the research that has got that? What is that based on? The conclusion is that drinking alcohol … especially breast cancer. What is the evidence for that? What is it based on?* (Group 1, ABC1)

*I don’t know, they bring out all these things, one thing is good for you, one ... next month there's something else that ... I think we go around in circles with things really* (Group 4, C2DE)

Understanding of the ActWELL programme as outlined in the invitation was good. Participants appreciated that the programme would provide personalised support for increasing activity, making changes to their diet, including alcohol intake, and setting goals in relation to their weight. The concept of a personal lifestyle coach was a familiar one and easily understood. Making changes to achieve a healthier lifestyle and manage their weight was generally an attractive prospect for most of the women interviewed. Some participants were already taking steps to increase their level of exercise, follow a healthy diet and manage their weight.

Third, it became apparent that it could be useful for coaches to acknowledge and reinforce attempts to change lifestyle. It was noted that participants commented regularly on attempts to increase physical activity. For example, one woman mentioned that she did not think she could become any more active in her life, given that she exercised on a near daily basis:

*I’m quite active every day, so I don’t know how much that would help. I go to … 5 mornings a week – swimming everyday I don’t think I could do anymore.* (Group 2, C2DE)

Fourth, this statement acted as a reminder that we must be clear that personalised advice is given to increase or maintain current physical activity. Where increases are recommended, these are based on current activity levels (however small or big) recognising that no one size (e.g. 10,000 steps) is appropriate for all. In addition, it is recognised that whilst there has been considerable emphasis on getting people more active, the importance of overall energy balance through dietary changes (reducing calorie intake) may have been underplayed - not least because weight management may be perceived more negatively. It is important to note that our target group are overweight or obese (BMI > 25 kg/m^2^) and will have a weight loss target that necessities an emphasis on dietary intake as the main factor.

There was a sense that ActWELL did not necessarily offer new information about how to achieve a healthier lifestyle or weight but rather it would provide the support, opportunity and motivation to make changes. Some were more sceptical about the support proposed by ActWELL. They questioned whether it was necessary to offer such a programme, highlighting that the information needed to make changes was already widely available and that those who wanted a healthier lifestyle could do this alone.

*We are responsible for our own health and wellbeing and if we’re interested enough there is plenty of information out there if you are interested in going to find things out.* (Group 1, ABC1)

These responses indicate that women can access health information, successfully act upon it and attain good health outcomes. However, as the programme targets overweight and obese women it is likely that these women will benefit from (further) lifestyle changes particularly in this age group where multimorbidity is common. One of the strongest features of the proposed programme is the combination of education with support and it is plausible that emphasising support (especially for women who have attempted weight change before) may be helpful in increasing engagement.

Finally, flexible timing of appointments appeared highly desirable. Participants appeared to have few issues with the structure of the ActWELL intervention or study processes. However, those who were in employment suggested that it would be better to have flexibility over the timing of appointments, with the ability to schedule appointments in the evening to fit around such commitments. Some commented that motivation would be better maintained with regular and relatively frequent contact with a lifestyle coach, given the length of time of the programme. Participants appeared satisfied with requirements to attend local hospitals for study or lifestyle coach appointments, though when leisure centres were suggested as venues some participants felt they were preferable, given issues with parking in hospitals and “clinical feel”. The changes made to the design of the full-scale trial in response to these findings are presented in Table 2 (row 2).

### ActWELL public advisory group

Most comments received were about recruitment approaches. Members highlighted that they were not experts on lifestyle change so did not comment on the specific targets of the programme. They did however comment on language and approach. Three elements that were noted as favourable were the sense of support, that help on behavioural change was being offered (and not demanded) and the activity “such as walking” (as opposed to “exercise”) were highlighted. In all communications, they highlighted that information on lifestyle should be sensitively communicated to avoid a perception of blame if cancer was diagnosed at a later point.

Consideration should be given to how the programme could be applied for people living with long-term disabilities who may be at higher risk because of current low physical activity levels. The changes made to the design of the full-scale trial in response to these findings are presented in Table 2 (row 3).

### Peer reviewer feedback

Most comments related to recruitment, analysis plans, measurements, avoiding cross-contamination and fewer comments on the actual intervention programme. The comments relating to the latter are as follows. Reviewers noted that the study was ambitiously powered for 7% weight loss at 12 months, highlighting the need to optimise delivery of the dietary component of the intervention to ensure best adherence (and weight loss) amongst the participants. They also noted that maintaining behaviour change over the longer term is challenging and requested details on strategies that might be implemented to assist with long-term adherence. Additionally, learning from the timing of the coaching sessions was queried (noting that in the feasibility trial, coaches had exceeded the planned time allowance) and to consider maximising coach adherence to the protocol. The changes made to the design of the full-scale trial in response to these findings are presented in Table 2 (row 4).

### Next steps

The feasibility intervention was designed largely around the use of behavioural change techniques but failed to consider how to incorporate more sustainable, long-term behaviour change strategies. The investigatory team highlighted the applicability of the COM-B model [22], which identifies the key components of capability, opportunities and motivation as determinants of behaviour change. The draft intervention was then reviewed, focusing on how these three aspects were being incorporated.

It was recognised that the draft intervention aimed to increase capability of dietary choices and weight management through personalised advice and practical, small changes (including portion size guidance). However, one of our previous studies [23] had increased capability on weight management through the provision of scales (for weekly weighing) and it was concluded these could be offered as part of the redesigned programme.

To increase opportunities for promoting physical activity we negotiated with local leisure centres to enable the face to face visits to take place in local premises, to offer a tour of facilities and to offer free/low-cost access to services. We also provided the participants with an information sheet on local walking and cycling groups within local areas.

Motivational techniques were used throughout the programme and specifically related to action to change behaviours principally through setting of modest and achievable goals, implementation intentions and the use of coping planning. The pedometers also provided potentially rewarding self-monitoring feedback of physical activity goals achieved.

Finally, a logic model was developed to identify how each component of the intervention programme was likely to moderate behaviours and impact on short-term and long-term behavioural outcomes and weight management. See Tables 3 and 4.

**Table 3:** Key components of the lifestyle coach sessions (face to face visits)

**Programme length**	12 months
**Contact**	Face to face
**Duration**	2 sessions - 60 min and 45 min, within 3 months
**Who delivers**	Trained lifestyle coach (volunteers from Breast Cancer Now)
**Place of delivery**	Local leisure centres (in office facilities)
**Professional support**	Telephone contact details provided
**Social support**	“Bring a buddy” offered, friend/partner/family member can be invited
**Theoretical framework**	COM-B model
**Summary of behaviour change techniques utilsed**	• Motivational interviewing • Goal setting (graduated/gradual, achievable) • Action plans (implementation intentions) • Coping planning • Self-monitoring and feedback
**Primary outcomes**	Changes in body weight and physical activity
**Action plans and implementation intentions**	Goals will be set for: • Weekly weight recording • Daily walking plan • Agreed food and drink (including alcohol) • Implementation intentions agreed (when, where and how)
**Coping plans self-monitoring**	Introduce activity focus • Provide pedometer • Pedometer/walking plan and diary • Offer body weight scales • Explanation of self-monitoring proceduresReview of previously set goals and modification, if necessary
**Education - breast cancer risk reduction**	5 min Evidence relating lifestyle breast cancer risk • Evidence on importance of lifestyle change after age 50 years • Further reading links • Brief background to which lifestyle factors increase risk and why - Weight - Physical activity - Alcohol • Weight gain and the risk of breast cancer • Reasons for eligibility (age and weight) and recognition that many women are already active and mindful of diet and body weight
**Education - physical activity**	20 min, including interactive walk and talk • Demonstration of brisk walking + pedometer (interactive) over a 10 min walk and talk session • Personalised walking plan (to fit with usual daily agendas) • Physical activity guidelines • Tips for decreasing sedentary behaviour • Links to a range of community opportunities provided • Introduction to leisure centre staff for access to premises (Set daily physical activity goal according to personalised walking plan)
**Education - diet**	45 min including interactive tasks (sugar in drinks/portion size quiz, dietary assessments procedures) • Drinks - the importance of water • Sugary drinks - sugar and calorie content • Alcohol - calories, alcohol, tips for cutting down, links for support • Snacks and discretionary foods - biscuits, chocolate, crisps, cheese • Meal patterns and healthy food choices (Eatwell guide) • Using traffic light labelling to guide food choices • Personalisation of eating plan (feedback on dietary assessment) • Importance of small changes and maintenance of these
**Education - weight management**	20 min including interactive task (personal identification of weight category, offer free body scales if required) • Discussion of goal to achieve (and maintain) 7% weight loss over 1 year using a 600 kcal energy deficit diet • Importance of diet and physical activity in weight loss • Personalised daily eating guide - according to body size, caloric requirements and food preferences
**Miscellaneous**	15 mins
	General support - listening re health, circumstances, experience of previous weight loss attempts. Non-judgmental approaches required at all times. Clarity that coaches are there to support not judge
	Coping plans (following illness, holidays, etc.)
	Getting family members involved for social support
	Agree future appointments to suit participant as far as possible

**Table 4:** Key components of lifestyle coach sessions (telephone calls)

**Contact**	Telephone (within 2 weeks after visit 1 and visit 2, then 7 calls over next 9 months)
**Time line**	Following on from face to face contact until 3 month follow-up assessment (6 calls total)
**Duration**	15 min
**Who delivers**	Lifestyle coach
**Professional support**	Make appointment for next telephone call
**Content**	General exchange about mental and physical health Elicit participant’s overview on progress and changes made Reinforce importance of modest behavior change for health benefit
	Discuss goals/restarts Discuss weight loss maintenance goals as appropriate - highlight ten top tips and habit progression
**Motivational approaches**	Discuss self-monitoring records Identify perceived diet/activity challenges
**Personal goals (implementation intentions)**	Continue to focus on implementation intentions and review these at next call Engage in coping planning e.g. reviewing previously set goals and modifying, if necessary
**Setting long-term goals**	Identify perceived achievements and summarise success Re-evaluate confidence, motivation and importance of changes made

### Final intervention programme

**Fig. 1:**
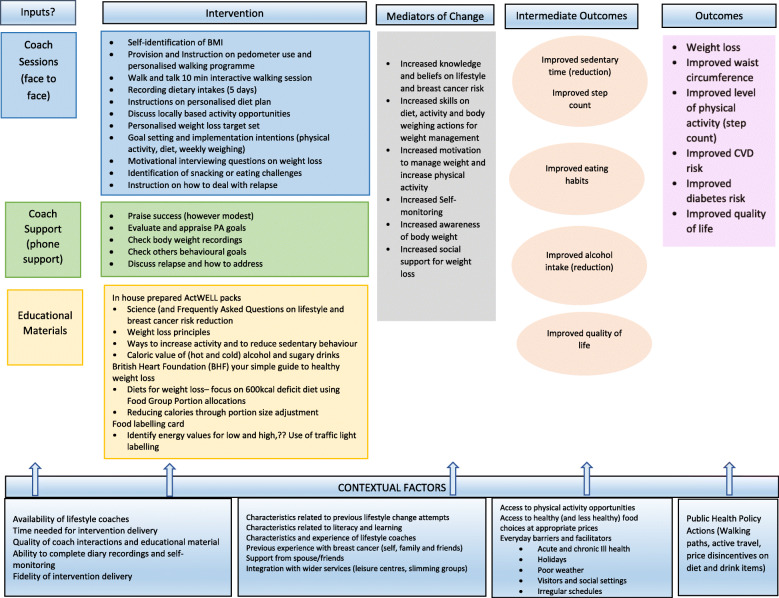
Logic model for impact of intervention

The intervention programme was finalised for delivery in two face to face sessions with nine support telephone calls. The key components of the face to face and telephone coaching sessions are presented in Fig. 1. Full details of the programme (including all written resources used) and trial details are provided elsewhere [24].

## Discussion

The ActWELL intervention programme has been developed to support weight management (weight loss and weight loss maintenance) based on evidence-based guidelines. The programme utilises the COM-B model to incorporate improved capability, opportunity and motivation for changing diet, drinks and physical activity in post-menopausal women. Lifestyle coaches combine educational approaches and behavioural change techniques in a non-judgemental and supportive manner to assist weight management over a 12-month period, to reduce breast cancer risk.

Identifying effective lifestyle interventions is a key part of comprehensive cancer control programmes with relevance for other non-communicable diseases [25]. Optimising interventions in content and delivery to motivate, initiate and maintain changes in behaviours and avoid unintended consequences is essential in the development of RCTs of complex interventions [15]. In the development and evaluation of RCTs for complex interventions designing, describing, and implementing a well-defined intervention have been described as the most challenging part of (undertaking) a trial of a complex intervention [26]. Where intervention trials have failed, it is unclear whether this is due to poor intervention design, failure to implement as planned or genuine ineffectiveness.

In a scoping review of 27 studies on optimisation procedures by Levati et al. [15] the authors concluded that methods for optimisation of interventions varied widely and there is no gold standard. Similarly, a recent systematic review of methods [27] to develop a taxonomy of intervention approaches identified eight different procedures. These ranged from partnerships with people who will use the intervention and interventions based on published research evidence, to stepped or phased approaches.

In recent years, frameworks to guide the optimisation of intervention design have been developed [28–30] and various modelling approaches identified [31], but there is an assumption that planning (and funding) of trials is linear and that timing will permit all stages of exploration to be undertaken. In reality, there is often no guarantee that funding for a full trial is available after feasibility work and if resources are obtained, the amount (including time) considered appropriate for intervention optimisation is often limited. Indeed, there may be concerns that multiple feasibility studies might need to be funded to improve indicative results and to ascertain when the intervention is “good enough”. In times of economic constraint, publicly funded researchers rarely have the luxury of extending funding timetables to allow development of the perfect intervention.

In the current work, the ActWELL feasibility study was used as the starting point for programme optimisation. However, there were some clear indications for change between the feasibility and main interventions that emerged during the optimisation process. The first was that body weight reduction (in women with BMI > 25 kg/m^2^) and physical activity were considered the most relevant primary outcomes to be targeted for cancer risk reduction. It is notable that these outcomes could be objectively self-monitored by participants with the potential to improve motivation. Second, most participants in the feasibility trial happened to have BMI > 25 kg/m^2^ (as anticipated from Scottish population data for women this age [32]) and this criterion became an eligibility requirement in the full trial. Third, partnership with Breast Cancer Now provided the opportunity for delivery by volunteer lifestyle coaches, which may prove more appealing, cost effective or sustainable than traditional weight management services. Finally, the intervention was previously delivered in research settings but the main trial utilised office facilities in local leisure centres.

The results from the feasibility trial justified progression to a full trial, and adjusting the programme and content to meet the new requirements provided an important opportunity for design input from the target group. The contributions provided significant direction for programme modification impacting on programme duration, coach contact time, content and use of behaviour change techniques as well as communications generally (e.g. science and evidence, non-judgemental approaches and avoiding guilt). In addition, the need for emphasis on support rather than education become very apparent.

The comments from peer reviewers focussed on the magnitude of effort required to achieve the intended weight loss and weight loss maintenance over a 1-year period. The intervention duration had not previously featured in the feasibility trial (12 weeks) given that the main aim of this was to assess practical trial issues, and participants had not been given the same ambitious weight change goals. This requirement is also challenging because of the absence of convincing evidence on how to support people beyond the intervention contact period. Our previous study of weight management in people diagnosed with a colorectal adenoma demonstrated continued weight loss between 3 and 12 months follow up by telephone contact, and it was anticipated that the same approach could again be successful [23].

The decision to use the COM-B framework for the intervention programme informed the focus on ways to facilitate change beyond our feasibility study. The extent to which these additional approaches have been utilised will be collected in post intervention interviews, in particular the use of leisure centre facilities and wider physical activity opportunities, which were highlighted in intervention materials. The programme utilises several behavioural change techniques successfully employed in the feasibility study. The techniques were originally chosen from a large range of possible approaches [33] because of their contribution to motivating and implementing change using techniques that can be delivered by trained lay staff.

What cannot be easily ascertained is the impact of individual parts of the intervention programme. Clearly, complex behavioural interventions contain multiple interacting components that challenge the identification of “active ingredients” per se. Our systematic process for optimising the programme now incorporates many promising components that can be delivered within the final ActWELL trial, which hold the promise of successful outcomes. Plausible mechanisms of how the components may act to influence behaviour have been identified within the logic model and each component of the intervention and mediators will be assessed, and feedback from participants attained, enabling greater insight into active components.

### Strengths and limitations

The current work aimed to optimise an intervention from feasibility to full trial for which there is no gold standard methodology. Our work started from a successful feasibility trial, which charities and government welcomed as a plausible action to reduce risk of post-menopausal breast cancer. Many of the approaches and techniques used had been previously used by the investigators and peer reviewers who were experienced in conducting successful weight loss trials. A significant number of the changes proposed have come from the public (from feasibility study, focus groups discussions and public advisory group). The latter involved women being asked to respond to a hypothetical intervention, so responses do not necessarily reflect how they would respond if they encountered the intervention in real life.

## Conclusions

The optimisation process has allowed the development of a programme that enables the delivery, testing and exploration of mechanisms of a promising intervention to achieve sustainable weight management in post-menopausal women, for use in a definitive randomised control trial.

## Data Availability

The datasets used during the current study are available from the corresponding author on reasonable request.
